# Developments in Deep Brain Stimulators for Successful Aging Towards Smart Devices—An Overview

**DOI:** 10.3389/fragi.2022.848219

**Published:** 2022-04-26

**Authors:** Angelito A. Silverio, Lean Angelo A. Silverio

**Affiliations:** ^1^ Department of Electronics Engineering, University of Santo Tomas, Manila, Philippines; ^2^ Research Center for the Natural and Applied Sciences, University of Santo Tomas, Manila, Philippines; ^3^ Department of Neurological Surgery, Davao Doctor’s Hospital, Davao, Philippines

**Keywords:** intervention for motor disorder, deep brain stimulation, DBS mechanism and hardware, open-loop DBS control, closed-loop DBS control, smart DBS

## Abstract

This work provides an overview of the present state-of-the-art in the development of deep brain Deep Brain Stimulation (DBS) and how such devices alleviate motor and cognitive disorders for a successful aging. This work reviews chronic diseases that are addressable via DBS, reporting also the treatment efficacies. The underlying mechanism for DBS is also reported. A discussion on hardware developments focusing on DBS control paradigms is included specifically the open- and closed-loop “smart” control implementations. Furthermore, developments towards a “smart” DBS, while considering the design challenges, current state of the art, and constraints, are also presented. This work also showcased different methods, using ambient energy scavenging, that offer alternative solutions to prolong the battery life of the DBS device. These are geared towards a low maintenance, semi-autonomous, and less disruptive device to be used by the elderly patient suffering from motor and cognitive disorders.

## Clinical Applications of Deep Brain Stimulation for Chronic Diseases

Deep brain stimulation (DBS) has developed during the past decades as a remarkable treatment option for several different disorders replacing ablative procedures (Lyons, 2011). There is a continuous expansion of the range of applications for deep brain stimulation (DBS) surgery since the initial observation of controlling or suppressing tremor with high frequency (130 Hz) thalamic ventralis intermedius (Vim) stimulation (Benabid, et al., 1996). With FDA approval, DBS has then been used for the therapy and management of certain chronic diseases such as Parkinson’s disease (PD) (Schupbach, et al., 2005; Koller, et al., 2000; Tani, et al., 2014), refractory or drug-resistant epilepsy (Salanova et al., 2015), dystonia (Hu & Stead, 2014), refractory essential tremors (ET) (Lyons & Pahwa, 2008), and dementia in Alzheimer’s disease (AD) and PD (Lv, et al., 2018).

Parkinson’s disease is an idiopathic, chronic, progressive and degenerative movement disorder that primarily affects the elderly caused by the progressive loss of striatal dopaminergic neurons in the substantia nigra (SNr) (DeMaagd and Philip, 2015). This upsets the balance between the direct and the indirect cortico-basal ganglia-thalamo-cortical (CBGTC) loop leading to its characteristic motor symptoms such as bradykinesia, resting tremors in several parts of the body, rigidity, and postural instability. Parkinson’s disease was uncommon before 50 years of age after which a notable increase in its prevalence with age was observed and peaked between 85 and 89 years (1·7% for men; 1·2% for women) and decreased after that age (GBD 2016 Parkinson’s Disease Collaborators, 2018). Up to 76–94% of PD patients appear levodopa-induced motor complications such as dyskinesia were considered for DBS therapy (Tran, et al., 2018).

Chronic epilepsy is a prevalent disorder that may be associated with significant abnormalities in cognition, brain structure, and psychiatric health that progress in some patients by middle age. It is associated with an increased prevalence of lifestyle factors associated with abnormal cognitive aging and dementia (Herman et al., 2008) and is characterized by spontaneous recurrent seizures and affects around 60 million patients worldwide, with 40% having drug-resistant epilepsy (DRE) (Engel, 2016). Prevalence of active epilepsy of idiopathic or secondary nature, for both genders, increased with age, with peaks at ages 5–9 years and at ages older than 80. The global age-standardized rate of disability adjusted life years (DALY) for idiopathic epilepsy was 182.6 for a population of 100,000 (GBD 2016 Epilepsy Collaborators, 2019). DALY is a summary measure of health loss defined by the sum of years of life lost (YLL). YLL peaked at age under 5 years and at ages of 15–19 years which then decreased progressively with age (GBD 2016 Epilepsy Collaborators, 2019). The years of living with disease (YLD) peaked at 5–9 years of age, decreased until 40–49 years, and increased progressively to the oldest age group (GBD 2016 Epilepsy Collaborators, 2019).

Dystonia is generally defined as a type of movement disorder with manifestations such as sustained or intermittent muscle contractions causing abnormal, often repetitive, movements, postures, or both. Dystonic movements are typically patterned and twisting, and may be tremulous. Dystonia is often initiated or worsened by voluntary action and associated with overflow muscle activation. This disorder was later classified by a consensus on movement disorders along two axes: clinical characteristics, including age at onset, body distribution, temporal pattern and associated features (additional movement disorders or neurological features); and etiology, which includes nervous system pathology and inheritance (Albanese, et al., 2013). Dystonia is poorly controlled solely by medication using anticholinergic drugs, dopamine modulators, pharmacologic agents, etc. Deep brain stimulation revolutionized its symptomatic treatment (Jankovic, 2013).

Tremor is generally defined as an involuntary, rhythmic, oscillatory movement of a body part. The original consensus criteria for classifying tremor disorders were published by the International Parkinson and Movement Disorder Society in 1998. A more updated criteria was later developed by Bhatia and others (Bhatia, et al., 2018) to account for subsequent advances in ET, tremor associated with dystonia, and other monosymptomatic and indeterminate tremors. The revised consensus statement classifies tremors along axes: clinical characteristics which includes historical features (age at onset, family history, and temporal evolution), tremor characteristics (body distribution, activation condition), associated signs (systemic, neurological), and laboratory tests (electrophysiology, imaging); and etiology (acquired, genetic, or idiopathic). Action tremors are classified as neurodegenerative (Aging-related tremors), and non-neurodegenerative (Essential tremors). Essential tremors constitute minor neurological findings such as mild cerebellar abnormalities which may either be hereditary (60–80%) and sporadic (20–40%) (Deuschl et al., 2015). Meanwhile, ARTs manifest as decline of aging parameters, including a change of cognition, activities of daily living, and reduction of strength and thereby a faster aging (Deuschl et al., 2015).

Dementia is the loss of cognitive functioning—thinking, remembering, and reasoning. One form of dementia is Alzheimer’s disease (AD) which is caused by changes in the brain, including abnormal buildups of proteins, known as amyloid plaques and tau tangle that aggravate with age. (https://www.nia.nih.gov/health/what-is-dementia). Table 1 summarizes these including the target section of the brain.

**TABLE 1:** Chronic diseases and corresponding DBS target sites.

disease	Target	References
Parkinson’s disease	GPi, STN, (PPN)	Deuschl et al., 2013; Deep Brain Stimulation for Parkinson’s Disease Study Group, 2001
Chronic Epilepsy	Cerebellum, CN, STN, hippocampus, CM, CC, LoC, MB)	Bergey et al., 2015; Fisher et al., 2010
Primary Dystonia	GPi, (STN)	Ostrem et al., 2011; Vidailhet et al., 2007
Essential Tremor	Vim, (STN)	Zhang et al., 2010; Blomstedt et al., 2010
Alzheimer’s disease	NBM, fornix	Laxton et al. (2010)

Abbreviations: GPi, globus pallidus internus; STN, subthalamic nucleus; PPN, pedunculopontine nucleus; CN, caudate nucleus; CM, centromedian nucleus of the thalamus; CC, corpus callosum; LoC, locus coeruleus; MB, mammillary bodies; Vim, ventral intermediate nucleus of the thalamus; NBM, nucleus basalis of Meynert.

## Efficacy of Deep Brain Stimulation for the Management and Treatment of Chronic Diseases

### Parkinson’s Disease

For the past several years, DBS has been established as a highly-effective therapy for advanced PD (Groiss et al., 2009), with options for treating PD symptoms continually expanding (Fox, et al., 2018). Based on an extensive evidence-based review conducted by the International Parkinson and movement disorder society, it was concluded that bilateral STN and GPi DBS are clinically useful for motor fluctuations and for dyskinesia when administered in tandem with the standard medications (Fox, et al., 2018).

On one retrospective analysis of the medical records of 400 consecutive patients who underwent DBS implantation, a 10-years survival rate of 51% for patients with PD has been reported using Kaplan-Meier estimation and multivariate regression utilizing Cox proportional hazards modeling (Hitti et al., 2019). The study results suggest that DBS provides durable symptomatic relief and allows many PD individuals to maintain activities of daily living (ADLs) over long-term follow-up exceeding 10 years. Meanwhile, a review paper and meta-analysis of eight eligible randomized control trials (RCTs) (n = 1,189) by Bratsos, et al. (2018), comparing the efficacy of DBS and best medical therapy (BMT) has shown that DBS provided more significant improvements based on the following outcome measures: Unified Parkinson’s disease Rating Scale (UPDRS), quality of life (QoL) using the Parkinson’s disease Questionnaire (PDQ-39), levodopa equivalent dose (LED) reduction, and rates of serious adverse events (SAE).

### Epilepsy

Deep brain stimulation has shown significant seizure frequency reduction on patients with drug-resistant epilepsy (DRE) across different age groups based from several independent studies as summarized in one review (Zangiabadi et al., 2019). In one follow up study investigating the long term efficacy of the clinical trial that involved the Stimulation of the Anterior Nucleus of the Thalamus for Epilepsy (SANTE), a median percent seizure reduction from the baseline for year one and year five was reported to be 41 and 69%, respectively (Salanova et al., 2015). Wille et al. (2011) reported 30—100% seizure reduction on five adult patients with progressive myoclonic epilepsy (PME) upon application of chronic high-frequency deep-brain stimulation.

### Dystonia

In one study comparing DBS with sham stimulation in a randomized, controlled clinical trial of 40 patients with primary segmental or generalized dystonia, it was shown that DBS has resulted in a higher movement score from baseline using the Burke–Fahn–Marsden Dystonia Rating Scale (Kupsch et al., 2006). The efficacy of continuous bilateral GPi-DBS was assessed on a prospective, controlled, multi-center study of 22 patients with primary generalized dystonia (Vidailhet et al., 2005). It was shown that after 3, 6, and 12 months of continuous bilateral GPi-DBS, dystonia motor symptoms were ameliorated by 47, 51, and 55%, respectively. Motor function has improved by 34, 42, and 44% at 3, 6, and 12 months, respectively based on the Burke-Fahn-Marsden Dystonia Rating Scale (BFMDRS). It was further shown that chronic bilateral pallidal stimulation is an efficient treatment option for patients with cervical dystonia who do not benefit from conservative treatment (e.g. local botulinum toxin injections) (Krauss, 2007); furthermore, there were significant improvements in dystonic posture and movements, reduced pain caused by dystonia and lesser related disabilities. Ostreem and Starr (2008) collated the different clinical trials on the application of DBS for dystonia treatment and has shown that, in general, significant improvement is manifested on patients with primary dystonia using BFMDRS.

### Alzheimer’s Disease

A review paper by Luo, et al. (2021), summarized 30 recent studies on the application of DBS to AD, 16 of which included actual clinical trials. On two independent studies, the memory of AD patients improved with the rate of cognitive decline decreased accompanied by an increase in cerebral glucose metabolism (Laxton et al., 2010; Smith et al., 2012). Other studies have also shown that the nutritional status of AD patients remained stable, and the rate of hippocampal atrophy slowed down after 1 year of DBS (Noreik et al., 2015; Sankar et al., 2015).

### Tremors

It was found that thalamic DBS is a safe and effective therapy in patients with essential tremor followed for up to 13 years based on the assessment done by Baizbal-Carvallo (2014). Here, 13 male patients (Age: 47 – 88 years) treated with DBS for essential tremor for at least 8 years were evaluated in the ‘on’ and ‘off’ state using the Fahn–Tolosa–Marin tremor rating scale, and their medical records were reviewed to assess complications related to this therapy. DBS provided a functional improvement of 31.7% in the ‘on’ state; furthermore, a total non-blinded improvement in the tremor rating scale of 39% was observed in the ‘on’ state. Meanwhile, on an observer blinded study of 20 patients with ET by Paschen et al., 2019, ventralis intermedius (ViM) DBS showed significant improvement over the non-stimulated condition based on the Tremor Rating Scale. However, it was further observed that Vim DBS loses efficacy over the long term (e.g. 10 years) for cases with medically refractory severe ET.

### Side Effects of DBS

Most DBS side effects can be understood as a result of current spreading into brain regions adjacent to the target area. Some of its common side effects include spastic muscle contractions, uni- or bilateral gaze deviation, autonomic side effects, *paresthesia*, speech impairment, *dyskinesia*, gait impairment and postural instability, acute neuropsychiatric side effects, depression, Impulse Control Disorders (ICD), and cognitive side effects (Koeglsperger et al., 2019).

## Mechanisms of Deep Brain Stimulation

Although DBS significantly reduces motor symptoms, limits drug-induced side effects, improves performance of activities of daily living, and enhances quality of life (Halpern et al., 2007), the corresponding physiological mechanisms are not fully explained (Montgomery and Gale, 2008). Several hypotheses offer an explanation on its mechanism namely: blockade depolarization, synaptic inhibition, desynchronization of abnormal oscillatory neuronal activity and antidromic activation (Li et al., 2014).

The blockade depolarization mechanism has been verified on an *in vitro* setup where high frequency stimulation can cause sustained depolarization of neural membranes by inactivating sodium channels and increasing potassium currents preventing the initiation or propagation of action potentials (Beurrier et al., 2001; Magariños-Ascone et al., 2002).

DBS is said to inhibit neuronal activity by reducing the firing rate of the neurons at the stimulated site similar to that of reversible lesion in ablative surgery (Herrington et al., 2016). This inhibitory activity was observed in normal awake monkeys where single-pulse stimulation of the GPi evoked brief inhibition in neighboring globus pallidus internus (GPi) neurons, mediated by the gamma-aminobutyric acid type A (GABA-A) receptors, while high-frequency stimulation of the GPi completely inhibited spontaneous firings of GPi neurons by activation of GABA-A and GABA-B receptors (Chiken and Nambu, 2013). This inhibitory activity was also observed intraoperatively on actual PD patients administered with STN-DBS (Filali et al., 2004; Welter et al., 2004), GPi-DBS (Dostrovsky et al., 2000; Lafreniere-Roula et al., 2010) and SNr-DBS (Lafreniere-Roula et al., 2010).

DBS is also said to be disrupting the abnormal flow of information in the cortico-basal ganglia-thalamocortical circuits (CBGTCs) during pathological conditions (Chiken and Nambu, 2016). Here, DBS activates axon terminals in the stimulated nucleus thereby inducing the release of inhibitory (GABA) and excitatory glutamate (Glu) neurotransmitters that dissociates the inputs and outputs in the stimulated nucleus. GABA is an amino acid released into the post-synaptic terminals of neurons that functions as the primary inhibitory neurotransmitter for the central nervous system (CNS). GABA causes hyperpolarization and inhibits neuronal activity. Glu, on the other hand, is an excitatory neurotransmitter. The neurotransmitter dopamine in the basal ganglia serves as the agent that modulates the functions of the striatum, the external and internal segment of the globus pallidus (GPe and GPi, respectively), the subthalamic nucleus (STN), and the substantia nigra pars compacta and reticulata (SNc and SNr, respectively) (Rommelfanger & Wichmann, 2010). The input and output nuclei of the basal ganglia are connected through two main pathways, i.e., the monosynaptic GABAergic “direct” pathway and polysynaptic “indirect” pathway. The latter involves GABAergic projections from the striatum to GPe and from GPe to the STN, as well as excitatory glutamatergic projections from the STN to GPe, GPi, and SNr. It was shown recently that nigrostriatal dopamine neurons inhibit striatal projection neurons by releasing a neurotransmitter that activates GABA-A receptors extending also to the mesolimbic afferents (Tritsch et al., 2014). Meanwhile, dopamine released from the striatum is also implicated in the modulation of learning and neuronal plasticity through processes such as long-term depression (LTD) or potentiation (LTP), acting at glutamatergic synapses (Pawlak and Kerr, 2008; Flajolet, et al., 2008). The balance between inhibitory neuronal transmission via GABA and excitatory neuronal transmission via glutamate is essential for proper cell membrane stability and neurologic function (https://www.ncbi.nlm.nih.gov/books/NBK526124/).

**FIGURE 1:**
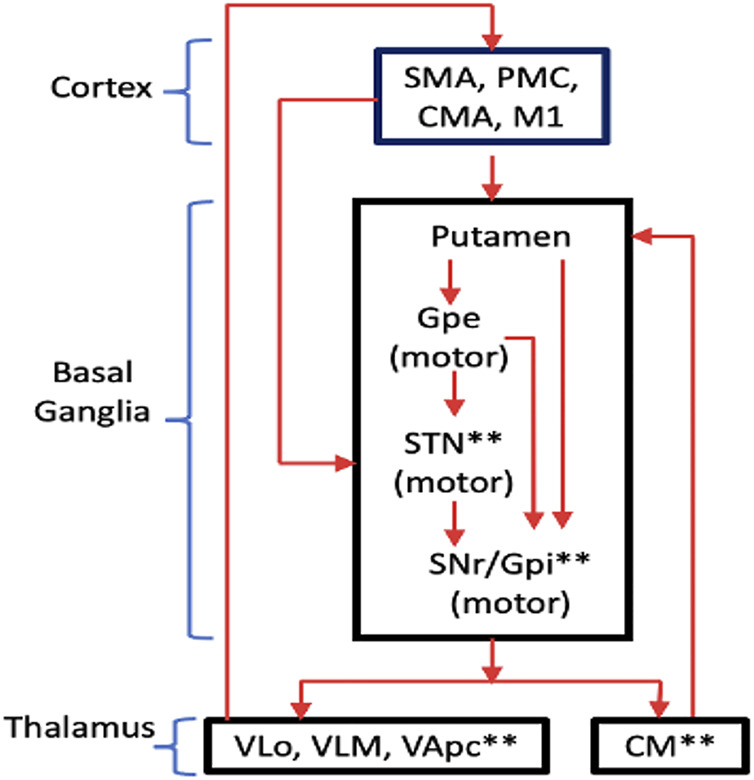
Motor circuit of the CBGTC showing the segregated sub-circuits. The targets of current DBS treatments are labeled with asterisks (*). Abbreviations: SMA, supplementary motor area; PMC, premotor cortex; CMA, cingulate motor area; M1, primary motor cortex; Gpe, globus pallidus externa; SNr, substantia nigra pars reticulata; Gpi, globus pallidus internus VLo, ventrolateral nucleus of thalamus, pars oralis; VLm, ventrolateral nucleus of thalamus, pars medialis; VApc, ventral anterior nucleus of thalamus, pars parvocellularis. (redrawn from Wichmann and Delong., 2011).

The basal ganglia consist of massive parallel and largely closed cortical-subcortical circuits, in which information is sent from different cortical areas to spatially separate domains of the basal ganglia where they are processed, and then returned to the frontal cortical area of origin via the thalamus (Wichmann and Delong, 2011). Based from the known functionalities of the cortical region, different CBGTCs may be classified as “motor,” “oculomotor,” “prefrontal,” (or “associative”) and “limbic” circuits. Each CBGTC is understood to consist of so-called “segregated” sub-circuits where the effect of DBS may be identified. Wichmann and Delong, 2011 showed an intuitive diagram of the motor circuit with its corresponding segregated sub-circuits as well as the DBS targets (Figure 1). Movement disorders, such as PD, dystonia and Tourette’s Syndrome (TS), are caused by dysfunctions in the motor circuit.

## Conventional Open-Loop Control Deep Brain Stimulation

**FIGURE 2:**
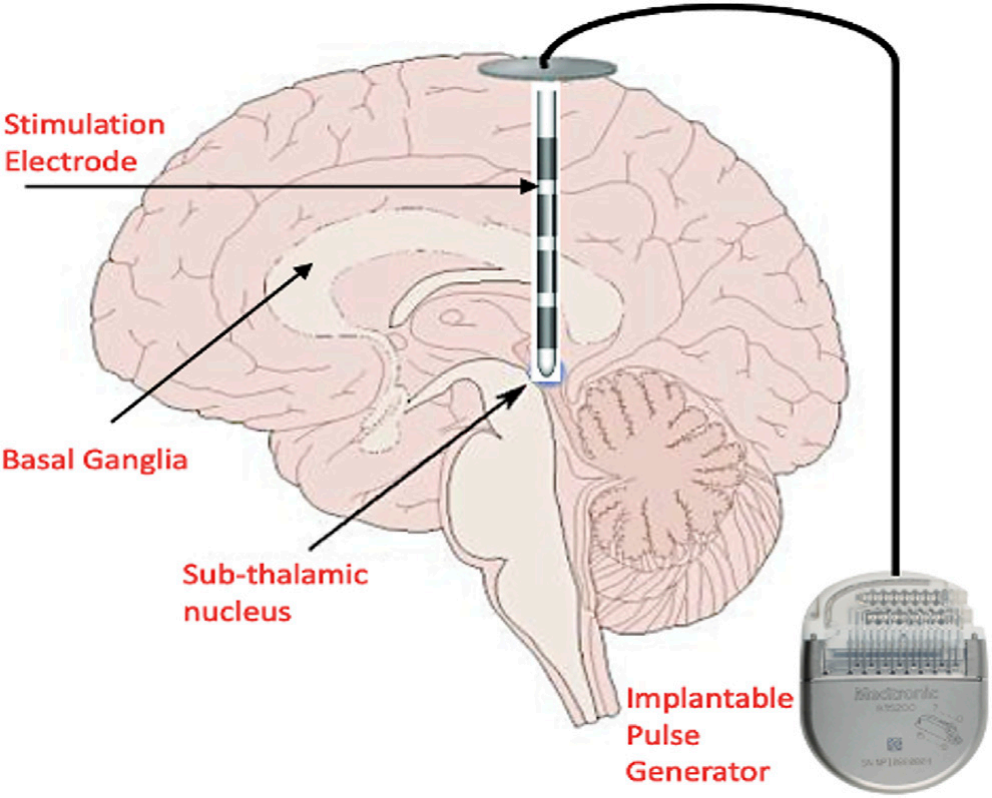
Conventional DBS setup showing the placement of the stimulation electrodes in the basal ganglia and sub-thalamic nucleus. The Implantable Pulse Generator (IPG) is placed in the sub-clavicular space, and extension wires are tunneled subcutaneously to connect the intracranial electrodes and IPG. The IPG is programmed remotely by the physician. (This figure indicates only cortical biomarkers and is a simplified diagram).

Harmsen and others (Harmsen, et al., 2020) consolidated the current state of affairs in the clinical trials for DBS registered in the Clinical-Trials.org database. The trials spanned 28 different disorders across 26 distinct brain targets, with almost 40% of trials being for conditions other than movement disorders. For addressing movement disorders, DBS is administered by implanting electrodes into any of the basal ganglia nuclei namely: GPi and STN (Halpern et al., 2007) and delivering pulses of preset amplitude, frequency, duration and polarity from an Implantable Pulse Generator (IPG) (Figure 2). Some of the typical DBS parameters used in disease management and therapy are summarized in Table 2.

**TABLE 2:** Typical DBS parameter settings.

Parameter	Value	References
Mode	• Constant Current (CC)	Fleming et al. (2020)
	• Constant Voltage (CV)	Stanlaski et al. (2012)
Amplitude	• CC: 0–3 mA	Fleming et al. (2020)
	• CV: 1–3.5 V	Stanlaski et al. (2012)
	• CV: 1–10 V	Wu et al. (2021)
Frequency	• Low Frequency (LF)	Su et al. (2018)
	- 60–80 Hz	Baizabal-Carvallo and Alonso-Juerez, (2016)
	- 20–45 Hz	Santaniello et al., 2011
	• High Frequency (HF)	Su et al. (2018)
	- 130–185 Hz	
Pulse Width	60–210 ms	Moro et al., 2002; O’Suilleabhain et al., 2003; Rizzone et al., 2001; Volkmann et al., 2002
Pattern of Stimulus	• Monophasic	Parastarfeizabadi and Kouzani, (2017)
	- charge imbalanced	
	• Biphasic	
	- charge imbalanced	
	- charge balanced	
	- passive	
	- active	
	- symmetric	
	- asymmetric	

Successful DBS depends on properly set stimulus parameters, including pulse width, frequency, and amplitude alongside with the proper electrode positioning (Su et al. (2018)). Determination of the optimal stimulation parameters is vital: to improve clinical efficacy; to minimize side effects; to maximize the battery life; and to evaluate the dose-response relationship between stimulation parameters and clinical effects. In one study by Obeso, et al. (2001), the final mean stimulus parameter settings that provided the highest efficacy to treat PD symptoms were 3V, 82 µs, and 152 Hz for STN-DBS, and 3.2 V, 125 µs, and 162 Hz for GPi-DBS. For the treatment of epilepsy, common DBS parameters are ≥100 Hz at 1–10 V for ANT stimulation for refractory temporal lobe epilepsy, ≥ 130 Hz at 1–5 V for hippocampus and STN stimulation for refractory temporal lobe epilepsy, tens to high frequency stimulation at 1–10 V for stimulation of centromedian nucleus (CMN) of the thalamus for generalized tonic-clonic seizures (Wu et al., 2021).

When finding the optimal DBS settings, the pulse width and frequency are initially kept constant at 60 μs and 130 Hz, respectively with gradual increase of stimulation amplitude in steps of 0.1–0.5 V or 0.1–0.5 mA until the safe treatment margin is obtained (Volkmann et al., 2006). Once the leads have been implanted stereotactically or via a surgical robot, each ring contact is tested in a monopolar configuration with the electrode as negative (cathode) and the IPG as positive (anode). Each of the rings or segments of the electrode are set to have the same stimulation intensity and are fired in unison (Volkmann et al., 2006). The mode of stimulation, either constant current (CC) or constant voltage (CV), has its corresponding pros and cons. CC stimulation provides a more precise control independent of brain tissue–electrode interface impedance variations but wastes significant amount of power and therefore reduces battery life, whereas, CV stimulation provides the reverse (Lettieri et al., 2015). The interface impedance tends to reduce post-operatively at an average rate of 73 Ω/year (Satzer et al., 2014). A recommended safe charge density limit of 30 mC/cm^2^ is normally considered in the selection of DBS parameters. Charge density is calculated by dividing the product of the voltage and the pulse width by the product of the impedance and the geometric surface area of the electrode (Kuncel and Gril, 2004).

The lack of understanding on the DBS mechanism makes the setting of stimulation parameters quite cumbersome. Several experimental studies, centered on PD, demonstrated that motor symptoms depend nonlinearly on the frequency and amplitude of stimulation (Moro et al., 2002; Moreau et al., 2008). Verification of DBS effects, i.e. STN-DBS for PD, is normally done by assessing rigidity, bradykinesia or (rest) tremor, and axial symptoms (Koeglsperger et al., 2019). Also, selected items from the Unified Parkinson’s disease Rating Scale (UPDRS) UPDRS or the Motion Disorder Society UPDRS (MDS-UPDRS) scale are used to assess the therapeutic effect and to document effects in a systematic manner.

To optimize therapy, a balance between maximal clinical improvement and minimal stimulation-induced side effects is being achieved through the adjustment of active electrode contacts, stimulus frequency, amplitude, and pulse duration (Mayo Clinic, 2017). This, however, is largely an ad hoc process that relies on clinical expertise and does not totally equate to optimal outcomes (Santaniello et al., 2011). Furthermore, the selection of parameters has important implications for power consumption, and thus the battery life of the implantable pulse generator (Santaniello et al., 2011; Parastarfeizabadi and Kouzani, 2017).

Conventional open-loop DBS involves the programming of the stimulation parameters based on the present condition of the patient. This is an iterative process in which stimulation parameters are adjusted to maximize therapeutic benefits while minimizing side effects (Morishita et al., 2013). However, the efficacy of these therapeutic parameters normally deteriorates over time due to disease progression, interactions between the host environment and the electrode, and lead migration (Grahn et al., 2014). Optimization of its efficacy is commonly achieved by multiple post-operative visits where the stimulation parameters are adjusted until the desired therapeutic effects are achieved with minimal adverse effects (Grahn et al., 2014). Risk factors abounding this process involve suboptimal outcomes, infections, device failure, and lead removal or repositioning (Frizon et al., 2019).

As a resolve, development of closed-loop control systems that can respond to variative neurochemical environments, tailoring DBS therapy to individual patients, is paramount for improving the therapeutic efficacy. This device is generally called “Smart DBS” because it is able to adapt dynamically to the condition of the patient and deliver the optimal electrical stimulation semi-autonomously (with minimal intervention) or autonomously.

## Closed-Loop Controlled Deep Brain Stimulation—“Smart” DBS

**FIGURE 3:**
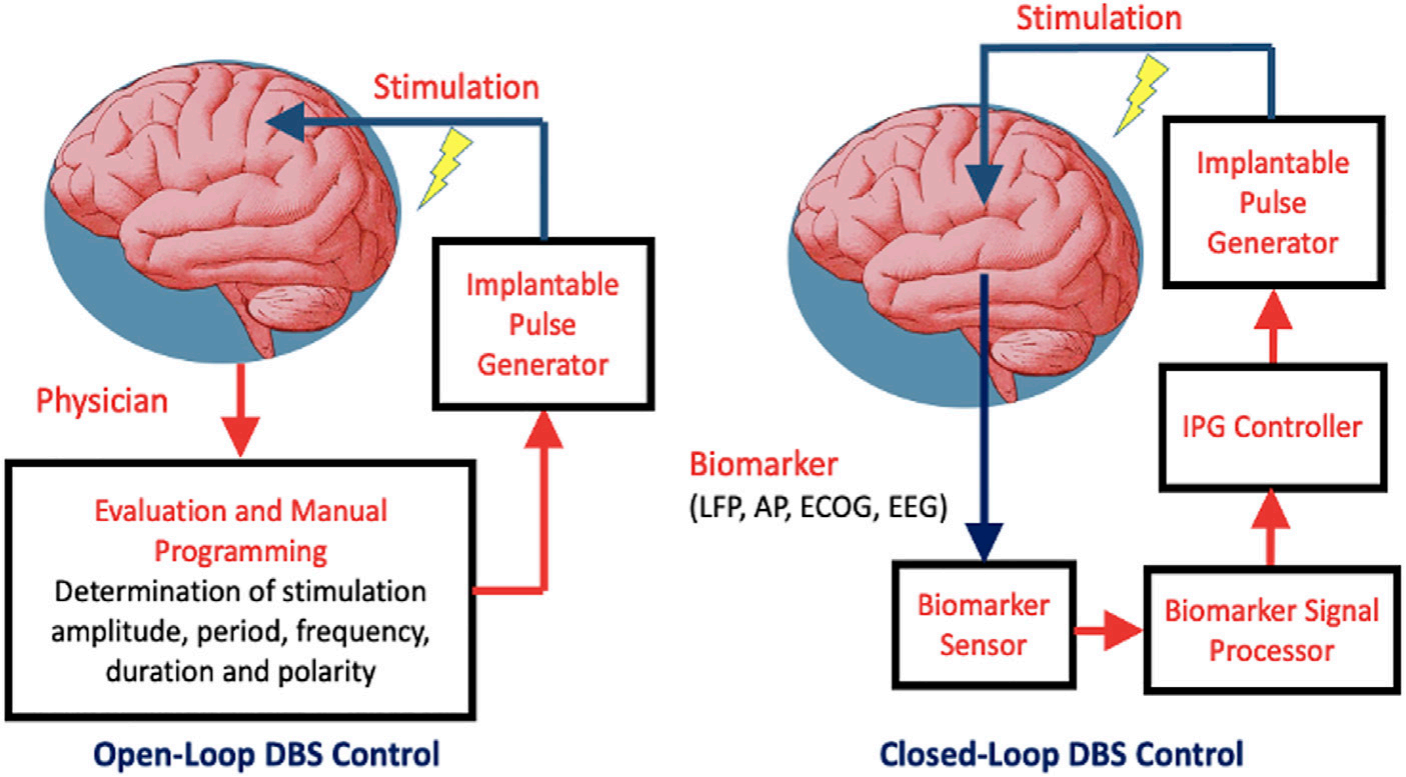
Two types of DBS control: open-loop and closed-loop. Abbreviations: IPG–implantable pulse generator; LFP–local field potential; AP–Action Potential; ECOG–Electrocorticography; EEG - Electroencephalography.

In a closed-loop DBS control, the clinical state of the patient is quantified periodically in order to adjust the stimulation parameters for optimal treatment while reducing stimulation induced side-effects (Fleming et al., 2020). The corresponding block diagrams of the DBS with open-loop and closed-loop controls are shown in Figure 3.

The DBS with closed-loop control consists of the neurofeedback loop where the stimulation is controlled either on/off or adaptively depending on the characteristics of a particularly biomarker. Such biomarker arises in lieu of a specific pathological condition. This loop is composed of biomarker sensor, signal processor, IPG controller and IPG device. Meanwhile, the DBS with open-loop control relies on the stimulation parameters programmed by the physician. Adaptive control involves dynamic adjustment of the stimulation parameters in response to the extent of the biomarker stimuli. In the presence of extensive pathological biomarkers, stimulation is prolonged with either its amplitude or frequency increased to deliver more stimulation energy, and vice versa. Meanwhile, to save on power, stimulation is deactivated whenever the preset biomarker threshold is not reached. Thresholding could either be singular or dual. The latter tends to be perform better in the presence of noise and offsets.

## Choice of the Biomarker for Closed-Loop DBS Control

**FIGURE 4:**
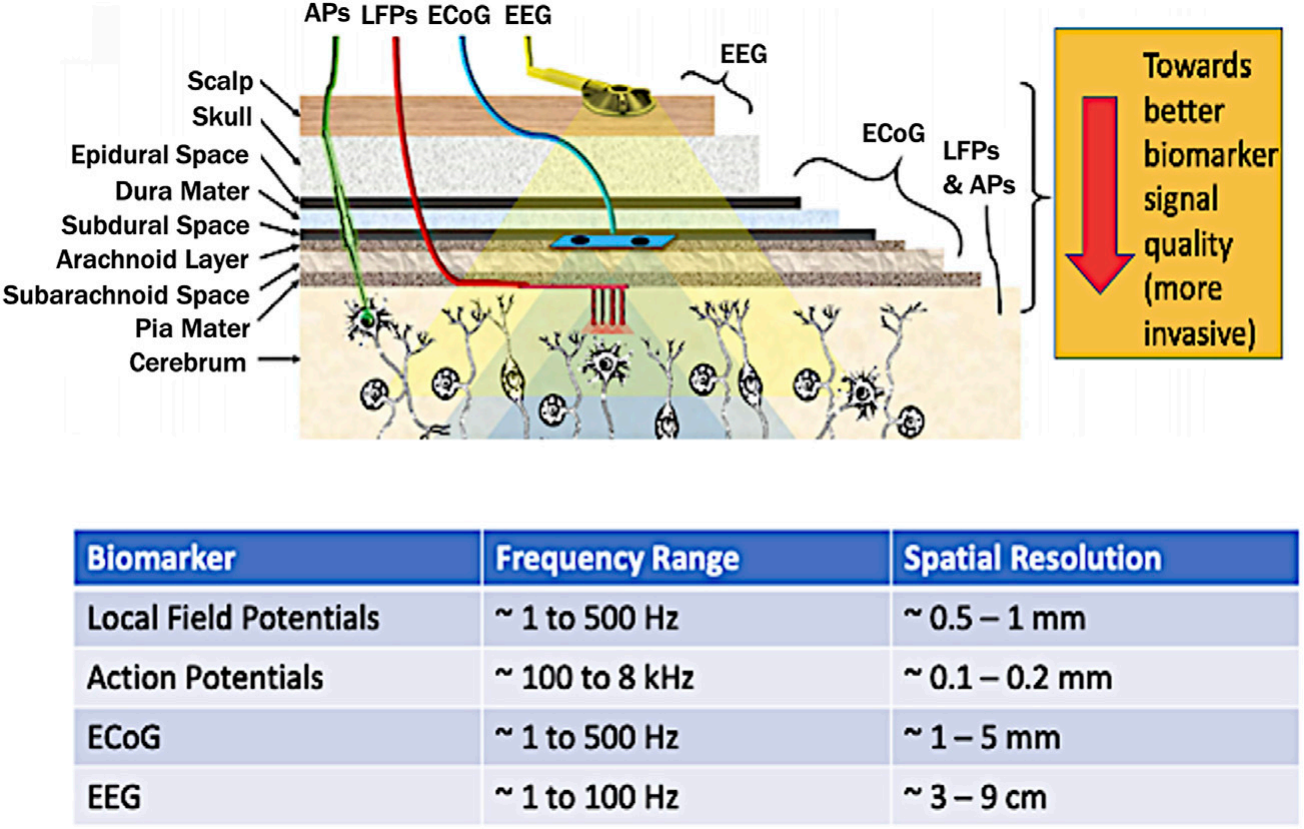
Brain biomarkers for closed-loop DBS stimuli (top figure adapted from Parastarfeizabadi and Kouzani, 2017). Abbreviations: LFP–local field potential; AP–Action Potential; ECOG–Electrocorticography; EEG - Electroencephalography.

To implement an autonomous or Smart DBS, the proper biomarker has to be identified. Several candidates have been considered in literature namely: electroencephalographs (EEG) (Abdelhalim et al., 2013), electrocortigraphs (ECoG) (Thomas and Jobst, 2015), Local Field Potentials (LFPs) (Abosch et al., 2012; Stanslaski et al., 2012; Little et al., 2013; Priori et al., 2013) and action potentials (Rosin et al., 2011) (Figure 4).

By considering the spatial resolution, proximity to the brain, and localization, LFPs are considered the most potent biomarker (Abosch et al., 2012; Little et al., 2013; Priori et al., 2013). Another key advantage is that LFPs can be directly recorded from the stimulation electrodes also achieving long-term stability at the electrode-tissue interface (Little and Brown, 2012). Meanwhile, other closed-loop control for DBS involved wearable sensors for detecting hand tremor (Sarikhani et al., 2019), and inertial measurement units (IMUs) for gait freezing (Bikias et al., 2021). However, for a fully implanted system which reduces the risk of infection, brain-based signals hold more ground since such system can be made in proximity with the stimulation electrodes.

The LFP is a summation signal of excitatory and inhibitory dendritic potentials from many neurons about the recording site. These are potentials generated in the extracellular space by propagation of APs through axons reflecting neuronal processes occurring within a local region around electrode in the neuronal extracellular space (Kajikawa and Schroeder, 2011). These have a spatial resolution of ∼0.5–1 mm (Schwartz et al., 2006), and frequency range covering ∼1–500 Hz with an amplitude of ∼200 µV (Einevoll et al., 2013).

It was observed that the energy signature of specific waves in the LFP signal, particularly the pathological beta waves (13—35 Hz), are directly related to abnormal brain activity associated to Parkinson’s disease (Rosin et al., 2011; Hariz, 2014; Hosain et al., 2014; Müller and Robinson, 2018). Hence, most works explore the energy of these waves as the biomarker for a potential closed-loop control DBS (Parastarfeizabadi and Kouzani, 2017; Müller and Robinson, 2018).

## Closed-Loop Control Schemes

Several controller models have been developed theoretically (Santaniello et al., 2011; Fleming et al., 2020). The controller of Santinello et al. (2011), was based on a recursively identified autoregressive model (ARX) of the relationship between the stimulation input and LFP output. It resulted to excellent performances in tracking the reference (tremor free) spectral features of the LFP through selective changes in the theta (2–7 Hz), alpha (7–13 Hz), and beta (13–35 Hz) frequency ranges, which is better than a static controller approach. In the work of Fleming et al. (2020), various closed-loop control algorithms *in silico* have been modeled incorporating extracellular DBS electric field, antidromic and orthodromic activation of STN afferent fibers, LFPs at non-stimulating contacts of the DBS electrode and temporal variation beta-band activity within the cortico-basal ganglia-thalamo cortical loop. The performances of various control modes such as on/off, dual threshold, proportional (P) and proportional-integral (PI) have been verified computationally, with PI yielding the optimum output in terms of power consumption and mean error in modulating the pathological DBS frequency. Meanwhile, the work of Molina, et al., 2021 demonstrated a closed loop DBS approach using bilateral GPi DBS implantation to address levodopa-responsive PD symptoms with open-loop stimulation, and PPN DBS to serve as feedback for the treatment of medication refractory Freezing of Gait (FoG). The primary outcome of the study was a 40% improvement in medication-refractory FoG in 60% of subjects at 6 months when "on".

## Hardware Implementations of Smart DBS

There have been several works that implemented the closed loop control either on an on-board module (Parastarfeizabadi et al., 2016; Parastarfeizabadi and Kouzani, 2017) or on a system-on-chip (Rhew et al., 2014; Wu et al., 2017; Wang et al., 2021). On-board module implementation involves the use of commercially available electronic components, microcontroller and digital signal processing modules. System-on-chip (SoC) implementations constitute miniaturized version of the DBS circuit blocks thereby providing a better form factor and less intrusive deployment than the on-board module.

### On-Board Module Smart DBS

A miniature closed loop deep brain stimulation device has been developed using dual energy thresholding for the on/off control (Parastarfeizabadi et al., 2016). The device incorporated pre- and post-amplifiers achieving 113 dB of gain, bandpass filter centered around 0.7–50 Hz, and a pulse generator, driven by a pico-power microcontroller unit, that provides on-demand stimulation current pulses of 90 µs duration, frequency 130 Hz, and amplitude 200 µA. Another work extended the DBS functionality to accommodate other diseases into one module (Parastarfeizabadi et al., 2016). This involved the neural sensor, a controller with a feature extractor, a 4 × 4 disease classifier using fuzzy logic, and a control strategy, and a neural stimulator. The front-end has a gain range of 50–100 dB, dual bandwidth of 7–45 Hz and 200–1000 Hz for the extraction of five biomarkers namely: five alpha, beta, sG, HFO, and spikes. The overall module dissipates 35 mW of power.

### SoC-Based Smart DBS Developments

System on Chip developments of the closed-loop DBS control have also proliferated. One work built a viable closed loop DBS SoC that utilizes logarithmic processing for the control and adaptation of stimulation currents based on detected low-frequency brain field signals (Rhew et al., 2014). Such method contributed to power savings while maintaining a wide dynamic range. Their system records and processes neural signals using four low-noise neural amplifier (LNA) channels, a multiplexed logarithmic ADC, and two high-pass and two low-pass digital logarithmic filters. A logarithmic domain digital signal processor (DSP) and PI-controller controls eight current stimulator channels and enables closed-loop stimulation. The SoC also incorporates an RF transceiver, a clock generator, and a power harvester. The overall SoC, implemented on CMOS 0.18 µm technology, has an overall area of 4 mm^2^ while consuming a total power of 468 µW for recording and processing neural signals, for stimulation, and for two-way wireless communication. Another SoC has been developed that incorporates a wireless power supply via an inductive link, a wireless interface, an adaptive high voltage tolerant stimulator, a bio-signal processor for seizure detection, and an 8-channel EEG acquisition unit (Wu et al., 2017). The acquisition unit consists of auto-reset capacitive-coupled instrumentation amplifiers (ARCCIA), band-pass filters, V-to-I programmable gain amplifiers, a multiplexer, a transimpedance amplifier (TIA), and a 10-bit DMSAR (Delta-Modulated Successive Approximation Register ADC). Its acquisition unit has achieved a Noise Efficiency Factor (NEF) of 1.77 with an input referred noise of 5.23µVrms, a stimulation current of 30 μA, and a standby power of 2.8 mW.

An 8-channel closed-loop neuromodulation SoC with 2-level seizure classification has been developed (Wang et al., 2021). It consists of a capacitive-coupled instrument amplifier (CCIA) at the analog front-end with a feedback-based common-mode (CM) cancellation circuit that suppresses large-scale CM interferences. Meanwhile, the stimulation artefacts are suppressed by a mixed signal loop. An auto-zero based pre-charge path boosts the input impedance, while the electrode DC offset is canceled by a DC servo loop with very-large and accurate time constant. The analog front-end chip occupies an area of 2.32 mm^2^ accompanied by a DSP with an area of 3.51 mm^2^. The CCIA can suppress 1.5-Vpp CM interference, and has achieved an accurate high-pass corner frequency as low as 0.1 Hz and an input impedance greater than 2.2 GΩ. The overall classifier achieves 97.8% sensitivity and consumes only 1.16-μW average power.

Another work on closed loop DBS control involved the two novel control algorithms for stimulator triggering namely: detection of gait arrhythmicity and logistic-regression model for the detection of gait freezing. Such controls were validated on a benchtop model in conjunction with a closed-loop DBS system by responding to real-time human subject kinematic and pre-recorded data from leg-worn inertial sensors from a participant with Parkinson’s disease. A novel control policy algorithm that changes neurostimulator frequency in response to the kinematic inputs has also been incorporated (O’Day et al., 2020). Another non-LFP based DBS control uses the hand tremors as input stimulus to trigger the implanted DBS module. Here, two sites of the basal ganglia (BG) namely the subthalamic nucleus (STN) and globus pallidus internus (GPi) are simultaneously controlled via stimulation using intelligent single input interval type-2 fuzzy logic (iSIT2-FL) combined with non-integer sliding mode control (SMC) (Gheisarnejad et al., 2020). On another work, neural sensing of movement (using chronically implanted cortical electrodes) was used to enable or disable stimulation for tremor. Therapeutic stimulation is delivered only when the patient is actively using their effected limb, thereby reducing the total stimulation applied, and potentially extending the lifetime of surgically implanted batteries (Herron et al., 2017).

### Commercially Available IPG Devices for DBS

Meanwhile, there exist some commercially available IPG devices for DBS with closed loop control features that have been successfully deployed clinically. One of which is the Activa™ RC + S system (Medtronic, Inc.) which records electrophysiological signals from the implanted DBS electrodes and offers inertial measurements (Hell et al., 2019). A more recent DBS system called the Percept™ PC platform (Medtronic, Inc.) incorporates “brainsense” technology utilizing LFP signals for refining therapeutic stimulation, symptoms tracking and correlation to neurophysiologic characteristics (Shahed, 2021). The Neuropace device has demonstrated responsive neurostimulation (RNS) and has been utilized for the treatment of drug-resistant epilepsy (DRE) (Shaikhouni, et al., 2015). A summary of the commercially available IPG devices for DBS is presented in Table 3 (Paff et al., 2020; Joohi, 2021; Shaikhouni, et al., 2015). It is noticeable that there are advancements in the features of IPGs such as rechargeability of the battery; multiplicity of the channels; wireless programmability and closed loop feedback. Meanwhile the efficacy of some of these commercial devices based on independent clinical studies are summarized in Table 4.

**TABLE 3:** Commercially available IPG devices.

Device	Frequency	Pulsewidth	Mode	Ampltiude (Joohi, 2021)	Feature	Application
Medtronic Activa™ PC (Paff et al., 2020)	2–250 Hz	60–450 µs	CC or CV	0–25.5 mA 0–10.5 V	conditionally safe with MRI	Bilateral STN and Gpi Stimulation for PD, Unilateral Thalamic Stimulation for Ets,Unilateral or Bilateral stimulation of the Gpi or STN for treatment of chronic, drug refractory segmental or generalized dystonia
Medtronic Activa™ RC (Paff et al., 2020)					dual channel, rechargeable, conditionally safe with MRI	
Medtronic Activa™ SC (Paff et al., 2020)	3–250 Hz				single channel, conditionally safe with MRI	
Medtronic Percept™ PC (Joohi, 2021)	2–250 Hz	20–450 µs	CC	0–25.5 mA	closed loop feature (using local field potential as biomarker)
Abbott Infinity 5 (Paff et al., 2020)	2–240 Hz	20–500 µs	CC	0–12.75 mA	dual channel	Bilateral STN and GPi stimulation for PD and for bilateral thalamic stimulation for ETs
AbbottInfinity 7 (Paff et al., 2020)						
Boston Scientific Vercise PC (Paff et al., 2020)	2–255 Hz	20–450—µs	CC	0.1–20 mA	dual channel	Bilateral STN stimulation for PD
Boston Scientific Vercise RC (Paff et al., 2020)			CC		dual channel, rechargeable	
Boston Scientific Gevia (Paff et al., 2020)					dual channel, rechargeable, conditionally safe with MRI	
PINS Medical G102 (Paff et al., 2020)	2–250 Hz	30–450—µs	CC or CV	0–25 mA;0–10 V	dual channel, remote wireless programming	PD, tremor, dystonia (Joohi, 2021)
PINS Medical G102R (Paff et al., 2020)					dual channel, rechargeable, remote wireless programming	
PINS Medical G101A (Paff et al., 2020)					single channel, remote wireless programming	
SceneRay 1180	1–1600 Hz	60–960 µs	—	—	dual channel remote wireless programming	—
Neuropace (Joohi, 2021)	1–333 Hz	40–1000 µs	CC	0–12.0 mA	closed loop feature (responsive neurostimulation), rechargeable (Shaikhouni, et al., 2015)	Drug-Resistant Epilepsy (DRE)

**TABLE 4:** Efficacy of some commercial IPG devices based on independent clinical studies.

Device	Study design	Disease	Test subjects	Efficacy	Scoring
Medtronic Activa PC + S (Molina, et al., 2021)	Interventional (clinical trial), single group assignment	Medication-refractory Freezing of Gait (FoG) in PD	5	40% improvement at 60% of the subjects after 6 months	FOGQ, PDQ-39 (Peto et al., 1998), GFQ (Giladi et al., 2000), ABC (Powell and Myers, 1995, UPDRS) (Fahn and Elton, 1987)
Boston Scientific™ (Moro, et al., 2010)	Nonrandomized, prospect, blinded, multi-center	PD	51	45.4% (STN), 20% (GPi)	UPDRS III
Boston Scientific™ Vercise System (Follett, et al., 2010)	Multi-center, randomized, blinded	PD	bilateral STN: 147; bilateral Gpi: 152	25.3% improvement in UPDRS III; improvement in 6 of 8 subscales	UPDRS III
Abbot St. Jude Medical INFINITY™ (Okun, et al., 2012)	Multi-center, randomized, blinded	PD	136	39% on the baseline UPDRS III scores improvement	UPDRS III
Medtronic Kinetra and Soletra (Schuepbach, et al., 2013)	Interventional (clinical trial), randomized, parallel assignment	PD	251	QoL improvement by 7.8 points	PDQ-39, UPDRS-II, III and VI

FOGQ, Freezing of Gait Questionnaire; GFQ, Gait and Falls Questionnaire; ABC, Activities Specific Balance Confidence Scale; PDQ, Parkinson’s disease Quality of Life Questionnaire, Unified Parkinson’s disease Rating Scale (UPDRS).

## Design Considerations for Smart DBS Implementation

Since the LFP signal is about 50–500 µV (Einevoll et al., 2013), the analog front-end that extracts the LFP should have low input-referred noise within the bandwidth of interest. However, solid state devices tend to generate a lot of noise especially in the frequency range of the biopotential signal which normally covers 0.5 Hz to 1 kHz (Ha et al., 2021; Parastarfeizabadi and Kouzani, 2017) The dominant noise in this spectrum is the flicker (1/f) noise which may be attributed to the crystal defects within the silicon material and silicon–oxide interface. The input referred rms noise voltages should be within <10 µVrms (Parastarfeizabadi and Kouzani, 2017). Corollary to this specification is the target signal-noise ratio (SNR) at the output of the AFE. An SNR of >40 dB is necessary to imply an intelligible signal. On the interface between the AFE and the tissue, several non-idealities exist namely: parasitic electrode impedance, ambient noise such as electromagnetic interference and power supply hum. To reduce these, the AFE should have a high common-mode rejection ratio (CMRR). This is defined as the ratio of the gain in the intensity of the intelligent signal (biopotential signal) over the gain of the common-mode signals resulting from the interface non-idealities and noise. A differential gain of >80 dB and a CMRR of >100 dB are considered typically for an AFE (Arlotti et al., 2016). The AFE should be able to reject large transients at the input and should accommodate a wide input dynamic range to prevent saturating its inputs. This is very essential since the DBS leads are shared for delivering the stimulation pulses and for extracting the LFPs. The AFE should be able to block the stimulation pulses while it is able to amplify the LFPs.

The stimulator should be programmable in amplitude (voltage/current), frequency, and in duration and phase. Different combinations of these parameters have been extensively used in clinical practice for different cases similar to PD. Generally, the stimulator should only be activated at defined intervals either based on demand (as in a closed loop case) or pre-programmed. This is to save battery life. A potential alternative or support unit for the embedded battery is an *in vivo* or a subcutaneous energy harvester. Several mechanisms for this have been explored in literature constituting mechanical energy, radio frequency, ultrasound, and thermal (Shi et al., 2018; Zhao et al., 2020; Zou et al., 2021). One work demonstrated the potential of harvesting ambient mechanical energy from pressure fluctuations in the CSF within the lateral ventricles of the brain (Beker et al., 2017). In general, the harvester should be designed to have the maximum efficiency possible and should be positioned where there are maximum physical stimuli while having minimal coupling loss. Other key considerations for developing these harvesters would be material biocompatibility, packaging, form factor, efficiency, and site practicality. For maximum power transfer, the harvester’s transducer should also be properly matched with the impedance of the front-end power scavenging electronics of the implantable device.

Finally, the overall power dissipation of a neural implant should be constrained so as not to cause any irreversible damage due to excessive current density and heating at the vicinity of the leads. To date, the power consumption of neural implants is within the range of 30 μW to 25 mW (Zhao et al., 2020), with most power attributed to the stimulator or to the wireless transceiver link.

Another aspect to consider in implementing a low maintenance DBS device is the need for supplemental energy sources that offer semi-perpetual charging with lower cost than present rechargeable devices. A typical rechargeable battery for DBS can support the device for a period of 9 years with an approximate long-term cost of care savings of $60,900 by considering lesser replacement surgeries, lesser number of clinical appointments and hospital visits, lesser need for preoperative planning, and lesser time off from work (Hitti et al., 2018). However, despite these advantages, a study conducted by (Khaleeq et al., 2019) showed that almost two thirds of patients with DBS, especially those who have a socially active and independent lifestyle, preferred the non-rechargeable IPG over the rechargeable ones. The choice is majorly because of convenience and concern about forgetting to recharge the battery. Furthermore, rechargeable DBS devices are more expensive than the non-rechargeable ones. According to the study of (Qiu et al., 2021), patients with less financial capabilities tend to choose the non-rechargeable DBS devices.

## Conclusion

In this overview paper, we have presented the efficacies of DBS therapy for diseases that aggravate with age based on independent clinical trials. We have also presented the current state of the art in DBS instrumentation, specifically the additive features of IPGs that cater for ease of use, monitoring, programmability and closed-loop control. Meanwhile, while such advancements are already on the market, innovation towards making the DBS therapy more stand-alone, semi-autonomous, and having smaller form factors are still underway. These specifically point to the developments in system-on-chip (SoC) implementations for closed loop control or “smart” DBS. This work detailed the future prospects of SoC-based DBS technology that tend to provide more freedom of movement and lesser intervention while highlighting its technical constraints and design challenges collated from technical literature. These can serve as guide for developing low maintenance DBS systems with an aim of improving the QoL of elderly patients.
